# Accuracy of pulse interval timing in ambulatory blood pressure measurement

**DOI:** 10.1038/srep37472

**Published:** 2016-11-23

**Authors:** Sarah A. Kane, James R. Blake, Frank J. McArdle, Philip Langley, Andrew J. Sims

**Affiliations:** 1 Medical Physics Department, Royal Victoria Infirmary, Newcastle upon Tyne, NE1 4LP, UK; 2 Institute of Cellular Medicine, Faculty of Medical Sciences, Newcastle University, NE1 7RU, UK; 3 School of Engineering, University of Hull, HU6 7RX, UK

## Abstract

Blood pressure (BP) monitors rely on pulse detection. Some blood pressure monitors use pulse timings to analyse pulse interval variability for arrhythmia screening, but this assumes that the pulse interval timings detected from BP cuffs are accurate compared with RR intervals derived from ECG. In this study we compared the accuracy of pulse intervals detected using an ambulatory blood pressure monitor (ABPM) with single lead ECG. Twenty participants wore an ABPM for three hours and a data logger which synchronously measured cuff pressure and ECG. RR intervals were compared with corresponding intervals derived from the cuff pressure tracings using three different pulse landmarks. Linear mixed effects models were used to assess differences between ECG and cuff pressure timings and to investigate the effect of potential covariates. In addition, the maximum number of successive oscillometric beats detectable in a measurement was assessed. From 243 BP measurements, the landmark at the foot of the oscillometric pulse was found to be associated with fewest covariates and had a random error of 9.5 ms. 99% of the cuff pressure recordings had more than 10 successive detectable oscillometric beats. RR intervals can be accurately estimated using an ABPM.

Automated blood pressure (BP) monitors provide a practical alternative to the traditional auscultatory method of blood pressure measurement and are widely used both in clinic and by patients at home. These monitors rely on the detection of the pulse at different applied pressures to estimate BP. Many manufacturers have developed BP devices which use pulse timings to detect irregularities of the heart beat, and some have incorporated algorithms for specific arrhythmias such as atrial fibrillation (AF), which have been shown to be both highly specific and sensitive^1^,^2^,^3^. An underlying assumption of this method is that the pulse intervals recorded using the BP cuff are an accurate representation of RR intervals. To our knowledge there are no studies demonstrating that this assumption is true. Validation of this assumption is important because incorrect pulse timing measurements could lead to misdiagnosis of arrhythmias and consequent distress for the patient and burden on the healthcare system.

The primary aim of this study was to determine the accuracy of pulse intervals derived from a BP cuff through direct comparison with the corresponding RR intervals. In particular we consider ambulatory blood pressure monitoring which is recommended by the UK National Institute of Health and Care Excellence (NICE) for the diagnosis of high blood pressure^4^ and involves taking multiple BP measurements. We aimed to measure the accuracy of pulse interval timings and to assess the effect of covariates (age, gender, mean arterial pressure, RR interval, cuff pressure and activity level). Our secondary aim was to estimate the proportion of ambulatory BP recordings which could be clinically useful for detecting AF.

## Methods

### Data Collection

Twenty healthy participants with no history of cardiac arrhythmia volunteered to take part in this study. Each wore a commercially available ambulatory blood pressure monitor (ABPM, Spacelabs 90217) for three hours. The ABPM was programmed to take a blood pressure measurement every 15 minutes throughout the study period. The monitor used a stepped deflation mechanism, i.e. it remained at a constant pressure until enough valid beats had been detected and then stepped to a lower pressure. If the ABPM determined that the measurement was invalid then it repeated the measurement two minutes later. Additionally, the participants wore a custom-built data logger which was pneumatically connected to the cuff of the ABPM and to standard ECG electrodes. This allowed synchronous measurement of both the pressure inside the BP cuff and the participant’s ECG (lead II) which were digitised and collected at a sample rate of 512 Hz. For each participant the initial measurement was made under controlled conditions in line with the guidance of the European Society of Hypertension^5^. The remaining measurements were under ambulatory conditions. Participants were asked to keep a diary indicating what they were doing at the time of each measurement (i.e. sitting/standing/walking). After three hours the ABPM and data logger were disconnected from the participant, concluding their participation in the study. The ethical aspects of this study were reviewed and approved by the Newcastle University Ethics Committee and informed consent was obtained from all participants. All methods were carried out in accordance with the relevant guidelines and regulations.

### Data Analysis

A measurement cycle was defined as a single period of cuff deflation resulting in a valid BP measurement. Each measurement cycle consisted of multiple pulse interval timing measurements. For measurement cycles where the ABPM was unable to make a valid BP measurement, the corresponding cuff pressure trace was excluded from the analysis.

The peaks of the R-waves in the ECG traces were identified using an algorithm developed in MATLAB^6^ which applied a threshold to the derivative of the ECG to locate R waves and then detected the local maxima to find the R-wave peak. Following the automated detection the positions of the R-wave peak locations were verified by one observer (SAK).

**Figure 1:**
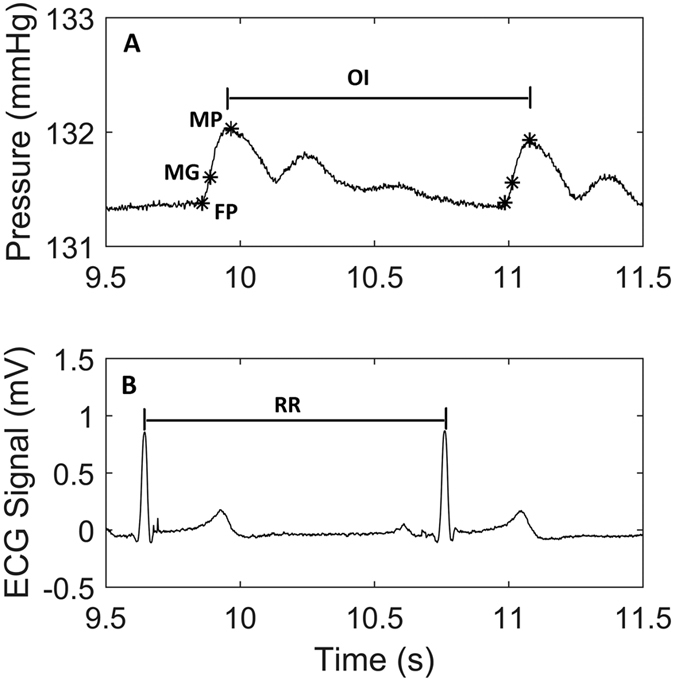
The oscillometric intervals (OI) were defined as the time interval between two consecutive landmarks of the oscillometric pulses in the BP cuff (**A**). These were compared with the corresponding RR interval timings (**B**).

The rising edge of each oscillometric pulse was identified manually by one observer (SAK). A fixed window was applied around each of these points within which the times of three oscillometric pulse landmarks (see Fig. 1) were automatically detected using MATLAB:

- Maximum point (MP) - the time of the maximum amplitude of the oscillometric pulse
- Maximum gradient (MG) - the time of the point of maximum gradient on the rising edge of the oscillometric pulse
- Foot of the pulse (FP) - an estimated onset time of the oscillometric pulse, found by the intersection of the tangent to the pulse at the point of maximum gradient and the tangent to the minimum point prior to the pulse.

If, during a single measurement cycle, one or more oscillometric pulses corresponding to an R-wave could not be visually detected, e.g. due to noise in the pressure trace, this cycle was classed as having an artifact. These recordings were included in the analysis however individual pulses were discounted if the oscillometric pulse could not be detected.

For each recording a series of RR intervals were measured from the ECG, and three corresponding sets of oscillometric intervals (OI) from the cuff pressure traces (one for each annotation landmark), Fig. 1. For each pulse interval the difference in timings between the two techniques was found by subtracting from each RR interval the corresponding OI (ΔRR = RR − OI). This gave three sets of ΔRR for each measurement cycle corresponding to the three annotation landmarks: ΔRR_MP_ , ΔRR_MG_ and ΔRR_FP_ .

#### Pulse Interval Accuracy

The ΔRR measurements were used to generate three mixed effects models using the *lme*4^7^ package in R^8^. The purposes of using mixed effects models were to account for repeated measures (multiple recordings per participant), unbalanced data sets (differing numbers of recordings per participant and numbers of beats per recording), fixed effects and random effects. The models were designed to determine the following:

1. if there was any systematic bias between the two methods (OI and RR);
2. an estimate of the random (experimental) error;
3. if there was any difference between the three annotation mechanisms (MP, MG and FP);
4. if any covariates influenced ΔRR.

The dependent variable in each model was ΔRR. Age (years), gender (M/F), mean arterial pressure (MAP, mmHg), cuff pressure relative to MAP (RCP, mmHg), RR interval (ms) and activity state (active/not active) were included as fixed effects in the model. A participant was classed as ‘not active’ during the measurement if they had recorded that they were sitting still during their measurement and classed as ‘active’ for any other activities (standing, walking, running etc.). The participant number and measurement number (nested within participant number) were included as random effects. Values for age, MAP and RR interval were centered to their median value for the whole population. The resulting models took the form of equation 1:


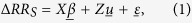


where *S* = {MP, MG, FP}, *X* is the design matrix for the fixed effects, *β* is a vector containing coefficients for the fixed effects, *Z* is the design matrix for the random effects, *u* is a vector containing the random effects and *ε* is a vector of the residuals (corresponding to the random measurement error). For each fixed effect coefficient 95% confidence intervals were determined using bootstrapping with 2000 simulations.

#### Clinical Utility

A potential use for the pulse timing measurements is to provide a method for opportunistic screening for AF. Many AF detection algorithms look for irregularities in pulse intervals and require a minimum number of successive beats. We therefore used the maximum number of successive oscillometric beats detected in a measurement cycle as a measure of the potential clinical utility of the recording. AF can be detected using pulse intervals in as little as 10 seconds using ECG recordings^9^ and BP monitors have shown high sensitivity and specificity for AF detection using only 10 successive oscillometric beats^10^ and so we used this value as a threshold to determine if a measurement cycle would potentially be clinically useful for AF screening.

## Results

**Figure 2:**
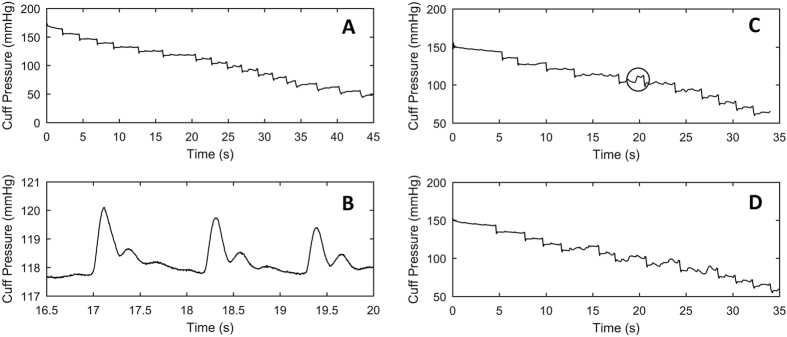
(**A**) An example of a cuff pressure trace obtained during the study. (**B**) An enlarged version of one of the pressure steps where three oscillometric pulses are clearly visible. (**C**) A trace with a small amount of movement artifact meaning that an oscillometric pulse cannot be identified (circled). (**D**) A trace with intermittent artifact throughout the entire recording.

Twenty healthy volunteers, aged between 24 and 61 years, were recruited. The range of valid BP measurement cycles was 9–13 and there were 243 valid BP measurement cycles in total. A total of 9677 R waves occurred during these measurement cycles. Oscillometric pulses were visible in all of the cuff pressure traces, Fig. 2. However, some cuff pressure traces were subject to artifact, Fig. 2. For the 9677 R waves, 8563 corresponding oscillometric pulses were visible meaning that for 1114 (11%) of the R waves no corresponding oscillometric pulse could be detected due to artifact. This left a total of 8093 RR and OI pairs available for analysis.

**Figure 3:**
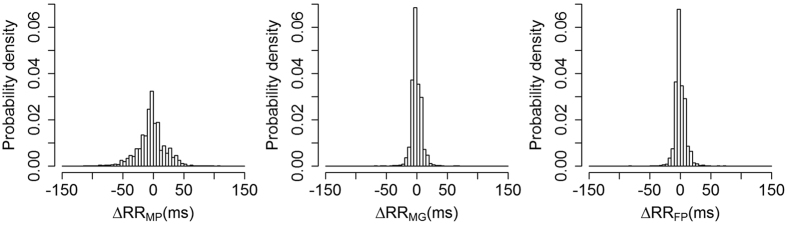
Distributions of ΔRR for each of the three pulse landmarks.

**Figure 4:**
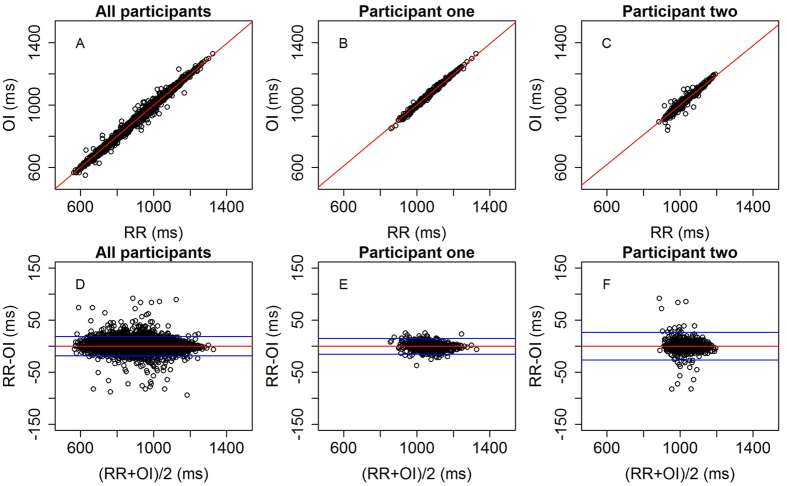
(**A–C**) Scatter plots of OI vs RR for the FP landmark for all participants (**A**, *R*^2^ = 0.997) and two individual participants (**B**, *R*^2^ = 0.995, (**C**), *R*^2^ = 0.976). Lines of best fit are shown in red. (**D–F**) Corresponding Bland-Altman plots. The central line shows the mean value of the difference and the upper and lower lines show the limits of agreement (mean ± 1.96*SD).

Figure 3 shows the distribution of ΔRR for each landmark. The MP landmark had the largest standard deviation of ΔRR at 27.3 ms. The standard deviation for both the MG and FP landmarks was 9.5 ms. Figure 4 shows scatter and Bland-Altman plots for the FP landmark for all pulses, for all participants, as well as for two individual participants.

### Pulse Interval Accuracy

**Figure 5:**
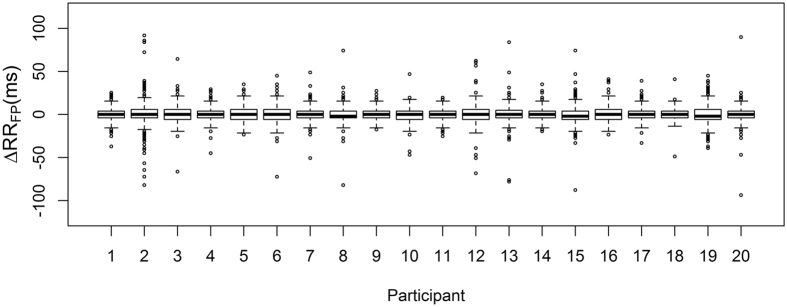
Distribution of ΔRR_FP_ for each participant. The boxes denote the inter-quartile range (IQR, upper: Q3, lower: Q1) of the data, the upper and lower whiskers are defined as min (max(ΔRR_FP_), Q3 + 1.5*IQR) and max (min(ΔRR_FP_), Q1-1.5*IQR) respectively. Outliers are any points outside of this range.

A summary of the results for the mixed effects models is given in Table 1. QQ plots and univariate plots for the models are given in supplementary Figures S1–S3. Age, MAP and RR interval were centred to the median values for the population (34 years, 90 mmHg and 912 ms respectively). The sizes of the random effects due to participant number and measurement order were small (less than 10^−6^ ms). This indicates that the between-participant variability is not significantly larger than the within-subject variability in the response as suggested by Fig. 5 for the FP case.

For each model the standard deviation for the residuals (which corresponds to the random measurement error) was less than 30 ms and was less than 10 ms for both the MG and FP landmarks (9.4 ms and 9.5 ms respectively). The MP landmark was the only method to have an intercept that is significantly different from zero, implying that there is a systematic bias associated with using this landmark (Table 1).

For the MP and MG landmarks, relative cuff pressure had a coefficient significantly different from zero. In this case the point estimates of the effect were −0.093 ms/mmHg and −0.025 ms/mmHg for MP and MG respectively. Over a typical measurement range of 100 mmHg, this is equivalent to a change in ΔRR of −9.3 ms and −2.5 ms over the course of a reading, approximately 1% or less of the median RR interval of 912 ms in both cases.

The MG and FP landmarks both had a non-zero coefficient for RR interval. In both cases the coefficients equated to a decrease in ΔRR of less than 0.004 ms per ms change in RR interval. If we consider this over the central 95% of RR intervals in this data set (691–1152 ms) this corresponds to a change in ΔRR of 1.84 ms, which is less than the error introduced by the sampling rate of the measurement (approximately 2 ms).

### Clinical Utility

**Figure 6:**
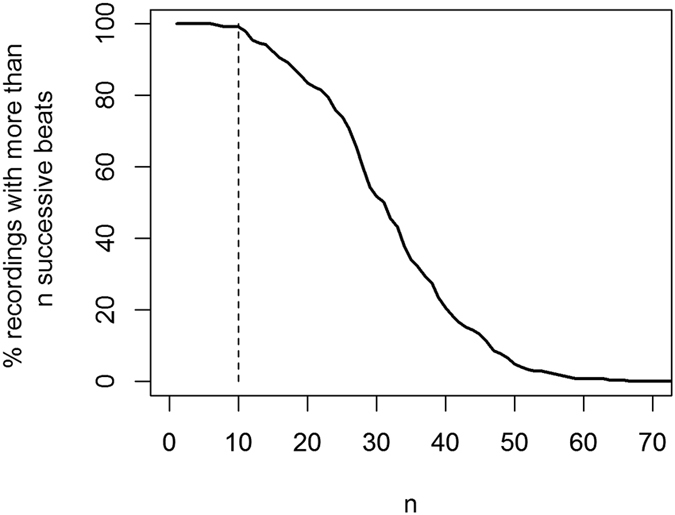
Number of measurement cycles with more than *n* successive beats in the recording.

Figure 6 shows the frequency distribution of the maximum number of successive detected beats in a single recording. Of the 243 measurement cycles, 241 (99%) had 10 or more successive detectable oscillometric beats in the cuff pressure recording and so have the potential to be clinically useful for AF screening.

## Discussion

We have directly compared pulse detection using an ambulatory BP cuff to R-wave detection using single lead ECG. Three different pulse landmarks were detected and the use of each to detect pulse intervals was analysed with a linear mixed effects model. One of the pulse landmarks (MP) gave a small (less than 2 ms) bias in ΔRR. For the remaining landmarks (FP and MG) statistically significant fixed effects were noted for cuff pressure (MG) and RR interval (MG and FP) though these are not considered to be clinically significant. Using the foot of the oscillometric pulse (FP) showed no measurement bias, minimised the effects of covariates and had a residual random error of less than 10 ms.

Many manufacturers have incorporated AF screening algorithms, which rely on pulse interval measurements, into their BP devices. These algorithms have been shown to have high diagnostic accuracy in both the clinical and at-home settings^11^. Accurate measurement of pulse intervals in the ambulatory setting could allow more opportunities for screening. Algorithms in clinical BP monitors require a minimum of 10 beats for AF screening^10^, 99% of our cuff pressure recordings had at least 10 successive detected beats. These results indicate that there may be scope to expand AF screening through blood pressure monitoring into this setting.

There are some limitations of this study. The work was performed on a small cohort of only twenty volunteers, though the mixed effects model demonstrated no participant effect on the pulse interval differences and so the results of these models should be generalisable. Additionally, all participants included in this study were in normal sinus rhythm and had no history of cardiovascular disease. If ABPMs are to be used for screening for AF then further validation will be required to determine if the results of this study hold for the target population in which there is a significant prevalence of hypertension, arteriosclerosis, valvular heart disease, coronary artery disease and heart failure. Arteriosclerosis is a degenerative stiffening of the arterial walls and hypertension also reduces the compliance of the arterial walls. These conditions are known to increase the velocity of the pulse wave through the arterial tree^12^. Heart disease will significantly change the volume and ejection velocity of the blood pumped into the arterial system during systole and, as discussed below, may change the shape of the pulse wave. However, for the purposes of using pulse intervals as a proxy for RR intervals, the important factor is to what extent the period between the ECG R-wave and the subsequent pulse recorded at the BP cuff can vary from one cardiac cycle to another.

The origin of the pulse is the pressure wave generated in the ascending aorta by the left ventricular stroke volume. The shape of the pressure wave in the ascending aorta is known to correlate sufficiently well with stroke volume^13^ meaning that devices based on pulse-contour analysis are becoming a popular means of monitoring cardiac output^14^. Conversely, the shape of the pressure wave will depend on the time course of the blood flow rate into the aorta during systole and so varies with stroke volume. Stroke volume varies with respiration^15^ and respiratory changes may be larger in an ambulatory setting than when sitting at rest. The shape of the pressure wave is further modified by its passage through the arterial system to the brachial artery and its transmission through the arm to the BP cuff, but we might expect beat-to-beat changes in the shape of the initial pressure wave to be reflected to some degree in the pulse observed in the BP cuff.

Pulse rate variability at the finger tips, measured using plethysmography techniques, can over-estimate heart rate variability as measured from the ECG^16^. Agreement is good for young, healthy subjects at rest but deteriorates with physical or mental stress often to an unacceptable level when the subject is physically active. Whilst it is acknowledged that motion artifact may contribute, physiological processes are also implicated. All three landmarks will be affected by beat-to-beat variations in the velocity of the pulse wave through the arterial system, but MG and MP may also be affected by beat-to-beat changes of the pulse shape. This may explain why FP was found to have the least variability in this study. Beat-to-beat variations in the velocity of the pulse wave will still affect FP, but the error in the measured latency of the pulse at the brachial artery will be considerably less than at the finger tips which are about three times as distant from the aortic arch.

Accurate estimation of pulse intervals using an ambulatory blood pressure cuff is possible. Using the time of the maximum point gave rise to a measurement bias. Using the maximum gradient or foot of pulse may lead to errors in the measurement of pulse intervals due to an association with cuff pressure (MG) or changing RR interval (MG and FP), though these are not likely to be clinically significant. Of the landmarks studied, for this population, the best choice of annotation landmark is the foot of the pulse, which minimised significant fixed effects and had a random measurement error of less than 10 ms. Ambulatory blood pressure recordings have the potential to be clinically useful for the detection of AF.

## Additional Information

**How to cite this article**: Kane, S. A. *et al*. Accuracy of pulse interval timing in ambulatory blood pressure measurement. *Sci. Rep.* **6**, 37472; doi: 10.1038/srep37472 (2016).

**Publisher's note:** Springer Nature remains neutral with regard to jurisdictional claims in published maps and institutional affiliations.

## Supplementary Material

Supplementary Figures

## Figures and Tables

**Table 1:** Results from the three linear mixed effects models investigating the effect of covariates on Δ*RR*.

	ΔRR_MP_	ΔRR_MG_	ΔRR_FP_
Random Effects (ms)			
Participant	<10^−6^	<10^−6^	<10^−6^
Measurement	<10^−6^	<10^−6^	<10^−6^
Residual	23.5	9.4	9.5
Fixed Effects (ms change in ΔRR/unit)			
Intercept (ms)	−1.715 (−2.596, −0.882)*	−0.131 (−0.449, 0.212)	0.005 (−0.331, 0.343)
Age (years)	−0.004 (−0.054, 0.046)	0.005 (−0.014, 0.025)	0.005 (−0.016, 0.024)
MAP (mmHg)	−0.010 (−0.078, 0.054)	−0.017 (−0.046, 0.009)	−0.003 (−0.029, 0.025)
RCP (mmHg)	−0.093 (−0.107, −0.078)*	−0.025 (−0.031, −0.019)*	0.000 (−0.006, 0.006)
RR interval (ms)	0.003 (−0.002, 0.008)	−0.003 (−0.005, −0.001)*	−0.004 (−0.006, −0.002)*
Gender (M/F)	0.165 (−1.154, 1.455)	0.492 (−0.043, 1.054)	0.256 (−0.275, 0.765)
Active (Y/N)	0.188 (−1.077, 1.379)	−0.083 (−0.587, 0.418)	−0.106 (−0.619, 0.343)

Results for the fixed effects are given as coefficient (95% confidence interval) and for random effects the standard deviation is quoted. * denotes a statistically significant result.
