# Supplementary material for: Accuracy of pulse interval timing in ambulatory blood pressure measurement
Source: Sci Rep. 2016 Nov 23;6:37472. doi: 10.1038/srep37472 (PMC5120306; doi:10.1038/srep37472)
Supplement: Supplementary Figures [file srep37472-s1.pdf]

Accuracy of pulse interval timing in ambulatory blood pressure measurement: supplementary material

Sarah A Kane[1,2*], James R Blake[1,2], Frank J McArdle[1,2], Philip Langley[2,3], and Andrew J Sims[1,2]

[1]Medical Physics Department, Royal Victoria Infirmary, Newcastle upon Tyne, NE1 4LP, UK
[2]Institute of Cellular Medicine, Faculty of Medical Sciences, Newcastle University, NE1 7RU, UK
[3]School of Engineering, University of Hull, HU6 7RX, UK
[*]sarah.kane@nhs.net

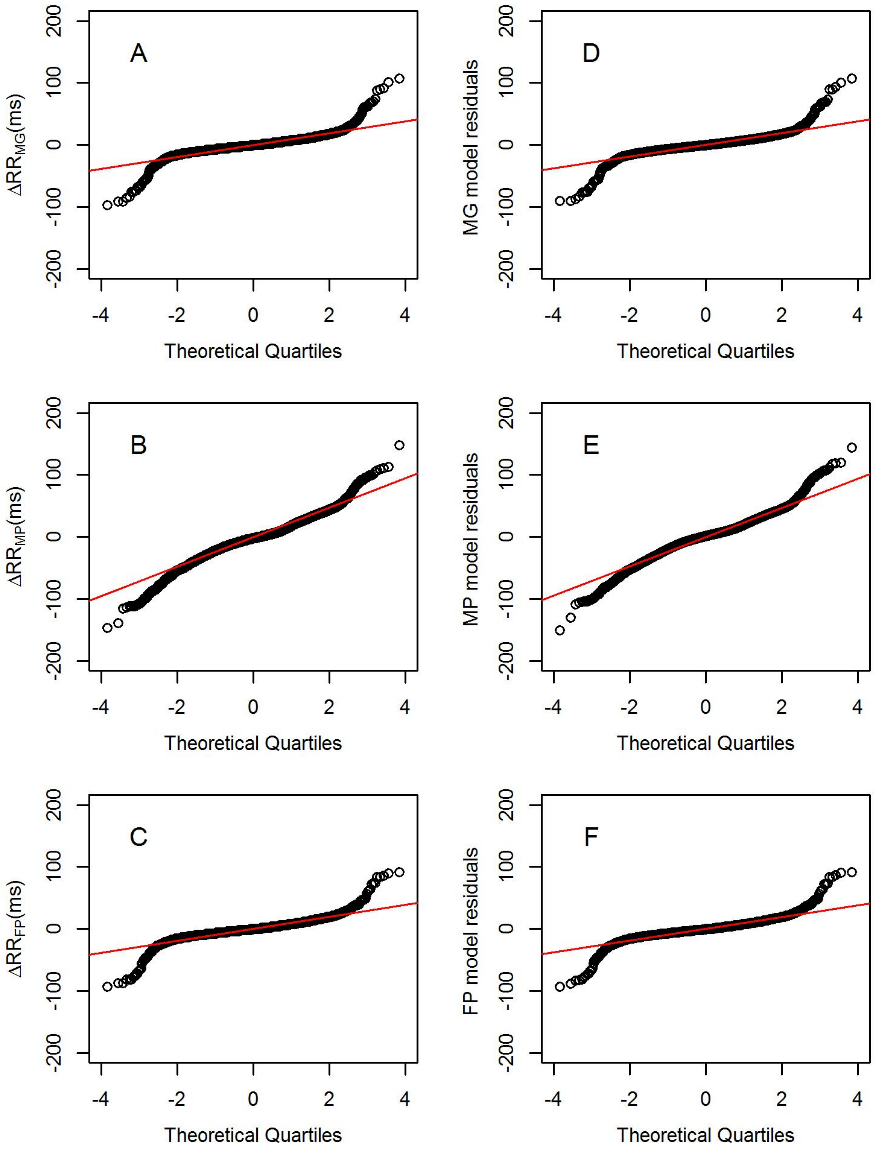

Figure S1. A-C: QQ plots for ΔRR for each annotation landmark
D-F: QQ plots of the residuals of the corresponding mixed effects models. The similarity between the corresponding distributions is consistent with the model results i.e. that fixed effects are not significant or are small.

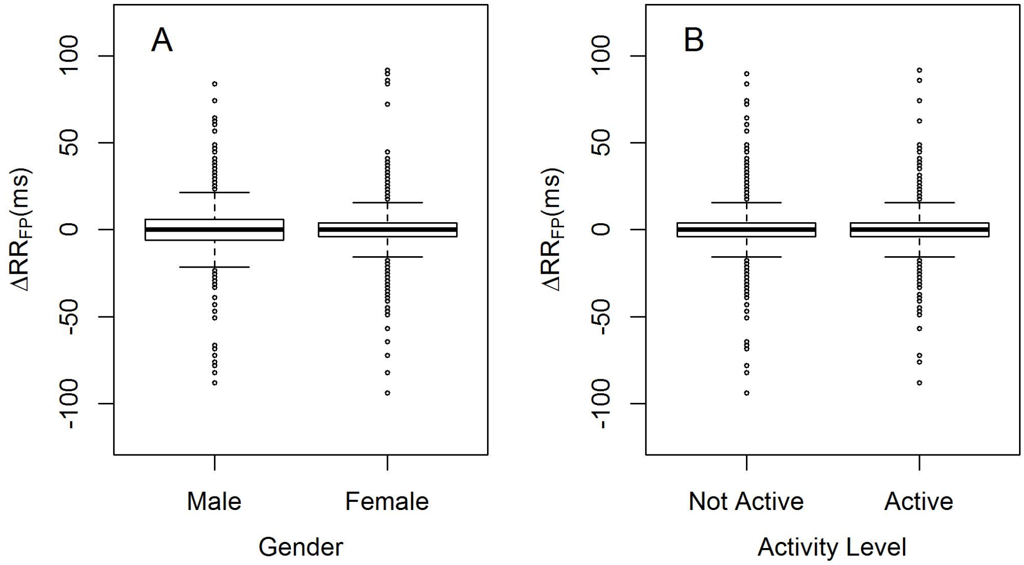

Figure S2. Box plots showing the distribution of ΔRR_{FP} based on gender (A) and activity level (B). The boxes denote the inter-quartile range (IQR, upper: Q3, lower: Q1) of the data, the upper and lower whiskers are defined as min(max(ΔRR_{FP}), Q3+1.5*IQR) and max(min(ΔRR_{FP}), Q1-1.5*IQR) respectively. Outliers are any points outside of this range.

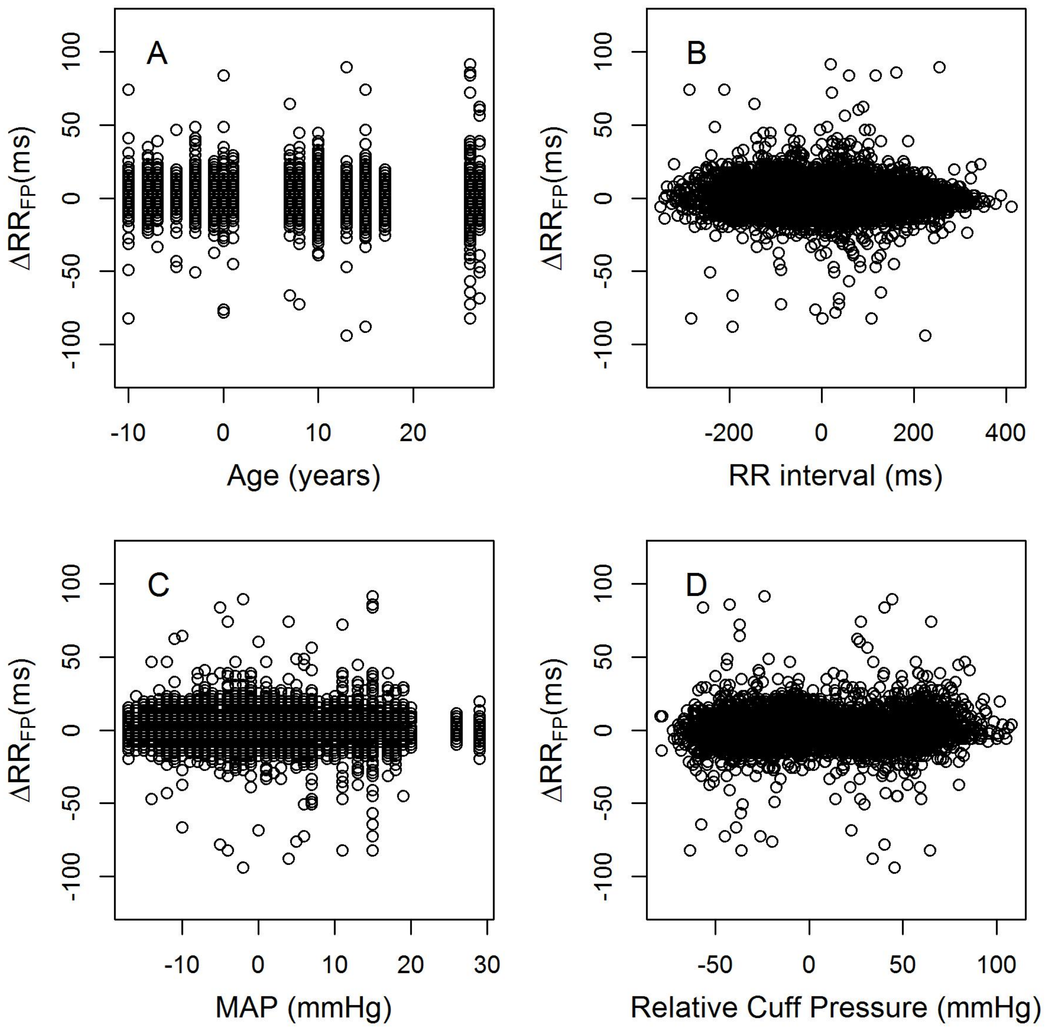

Figure S3. Univariate scatter plots for fixed effects included in the model
A: Age, centred to the median value for the population (34 years)
B: RR interval, centred to the median value for the population (912 ms)
C: Mean arterial pressure, centred to the median value for the population (90 mmHg)
D: Cuff Pressure relative to the mean arterial pressure for the recording.
